# A comparative study of red brick powder and lime as soft soil stabilizer

**DOI:** 10.12688/f1000research.27835.1

**Published:** 2021-08-09

**Authors:** Aisyah Salimah, Miftah Hazmi, Muhammad Fathur Rouf Hasan, Putera Agung Maha Agung, . Yelvi

**Affiliations:** 1 Civil Engineering, Politeknik Negeri Jakarta, Depok, Jawa Barat, 16425, Indonesia; 2 Directorate General of Water Resources, Ministry of Public Works and Housing, Jakarta, DKI Jakarta, Indonesia

**Keywords:** Soft soil, lime, brick powder, CBR, hambalang

## Abstract

**Background:** Soil has an important role to play in planning buildings because it supports the loads above it. Different types of soil with poor mechanical properties require more attention. Therefore, it is necessary to put in more effort to stabilize soil in order to improve its properties. This study aimed to compare the potential of lime and brick powder as stabilizers based on the values of California bearing ratio (CBR). Soil stabilization can be defined as the process of stabilizing soil properties by chemical or physical means to improve its engineering efficiency. The main objectives of stabilizing soil are to increase the bearing capacity of soil, to increase its resistance to weathering processes, and its permeability.

**Methods:** In this work laboratory tests were done with disturbed and undisturbed soil samples. The proportions of lime or red brick powder additives mixed together are 0%, 5%, 10%, and 15% of the original soil sample. From the results of the laboratory tests, the soil type obtained is MH soil based on the Unified Soil Classification System (USCS). The MH soil type is a low plasticity silt soil.

**Results:** The study’s results showed that with the addition of lime and brick powder, the soil could be stabilized. In both soaked and unsoaked CBR tests, there was an increase in the CBR value for each proportion of the mixed additives. However, red brick powder had a significant increase of 15%.

**Conclusions:** This study found a very large range of variations because of the many material requirements for each test. We suggest other researchers perform the CBR test by reducing the range of variations in the additives to get firm data and using our experimental procedure in this study for further research.

## Introduction

Soil is considered to be a three-phase system consisting of soil particles, pores (air) between its particles, and liquid (water) which varying degrees, fills and flows through the pores. ^1^ Based on the particle size of soil, there are several types of soil, namely gravel, sand, silt, clay. ^2^ The large content of silt and clay in soil affects its geotechnical characteristics that can vary: shrink when dry, and expand when wet; in the presence of water, they swell and become plastic. ^3^ Constructions such as buildings, highways, bridges, tunnels, dams, and towers are established on the ground that functions to support the loads above it. ^4^ Engineers often have problems using soft soil, which has; weak mechanical properties. Soft soil is a cohesive soil consisting of very small grains, characterized by low shear strength and high compressibility, which does not possess sufficient strength to support loads. It is necessary to treat these soils to provide a stable subgrade and avoid excessive land subsidence. A soil is categorized as soft soil if its shear strength value is 12 – 25 kPa. ^5^

For these reasons, soft soils need treatment before they can be used as a material subgrade by enhancing their engineering properties. Soil stabilization aims at improving soil properties and increases its resistance to softening by water. In principle, it means rearranging soil grains for them to be very tight and interlocked together. ^6^^,^^7^ The stabilization process, which is mixing soil with additives, can change the texture or plasticity of soil, its gradation, or act as a binder for soil cementation. ^8^

In recent years, a considerable number of field and laboratory experiments have been carried out using various additives, such as lime, ^9^^–^^11^ silica fume, ^12^ and fly ash. ^13^^,^^14^ However, not much research has been done on soil stabilization using red brick powder as a soil stabilizer. ^15^^,^^16^ Moreover, every red brick manufacturer knows its history, which does not always have the same characteristics. This could indicate differences in the test results at every location. ^17^ Therefore, it is necessary to do more research on the effect of red brick powder on soil stability. Also, lime and red brick powder are compared in this work. The impact of lime and red brick powder on the geotechnical qualities of soft soil was examined by conducting the CBR test. The CBR values for the soil mixture progressively increased. ^18^^,^^19^ The CBR value increased because the soil structure changed from being dispersed to flocculated. Thus, in this study, lime and brick powder were used as stabilizers on soft soils.

The study aims to compare the potential of lime and brick powder in stabilizing soft soil. Soil stabilization parameters were measured based on their effect on the CBR values of soaked and unsoaked soil samples *.* This test is a penetration test which entails inserting an object into the test object. Through this way, the strength of the base or other materials used to make the pavement can be assessed. As soil is not always in a dry condition, it would not be enough to do the CBR test with unsoaked soil samples; it must be done also with soaked soil. Soaking simulates adverse moisture conditions such as those caused by possible rain or flooding, and it is used in most CBR test. The difference between the soaked and unsoaked CBR testing procedure is that in the soaked CBR, the soil sample that has been molded is first soaked for 4 days (96 hours) by placing a standard load of 10 lb above the mold and then penetration test is carried out afterward. The mixed proportions of lime or red brick (additives) powder are 0%, 5%, 10%, and 15% of the original soil. In this study, the index and engineering properties of the original soil and mixed soil were tested. Index properties test includes testing moisture content, specific gravity, Atterberg limit, and grain size analysis. While testing the original soil with a variation of the mixture of lime or red brick powder, the standard compaction test and CBR analysis are carried out.

### Literature review

Lime is commonly used as additional material for soil stabilization especially for the construction of highways. Lime reacts with soil and changes its mineral properties. This is due to its reaction with calcium ions, which leads to the formation of cementitious holding capacity of soil (decreases moisture content), reduction of swelling, and improvement of soil stability. ^20^ Previous studies ^21^^,^^22^ revealed that the stabilization of subgrades by lime could significantly improve their engineering properties. Using lime for soil stabilization gives a significant level of long-term strength gain, which is developed through a long-term pozzolanic reaction. ^23^

Red brick is made of clay, which is burned at a high temperature until a reddish color is obtained. It cannot be broken down when immersed in water. Red brick has refractory properties and can withstand compressive loads. ^24^ There are scanty research works that have used red brick powder as a stabilizer because it is not commonly used. Each area has different red brick characteristics. Previous researches works have found that in CBR results, the primary strength parameters of soil were substantially improved by the use of brick powder. ^25^

## Methods

### Material and sampling

**Figure 1.**
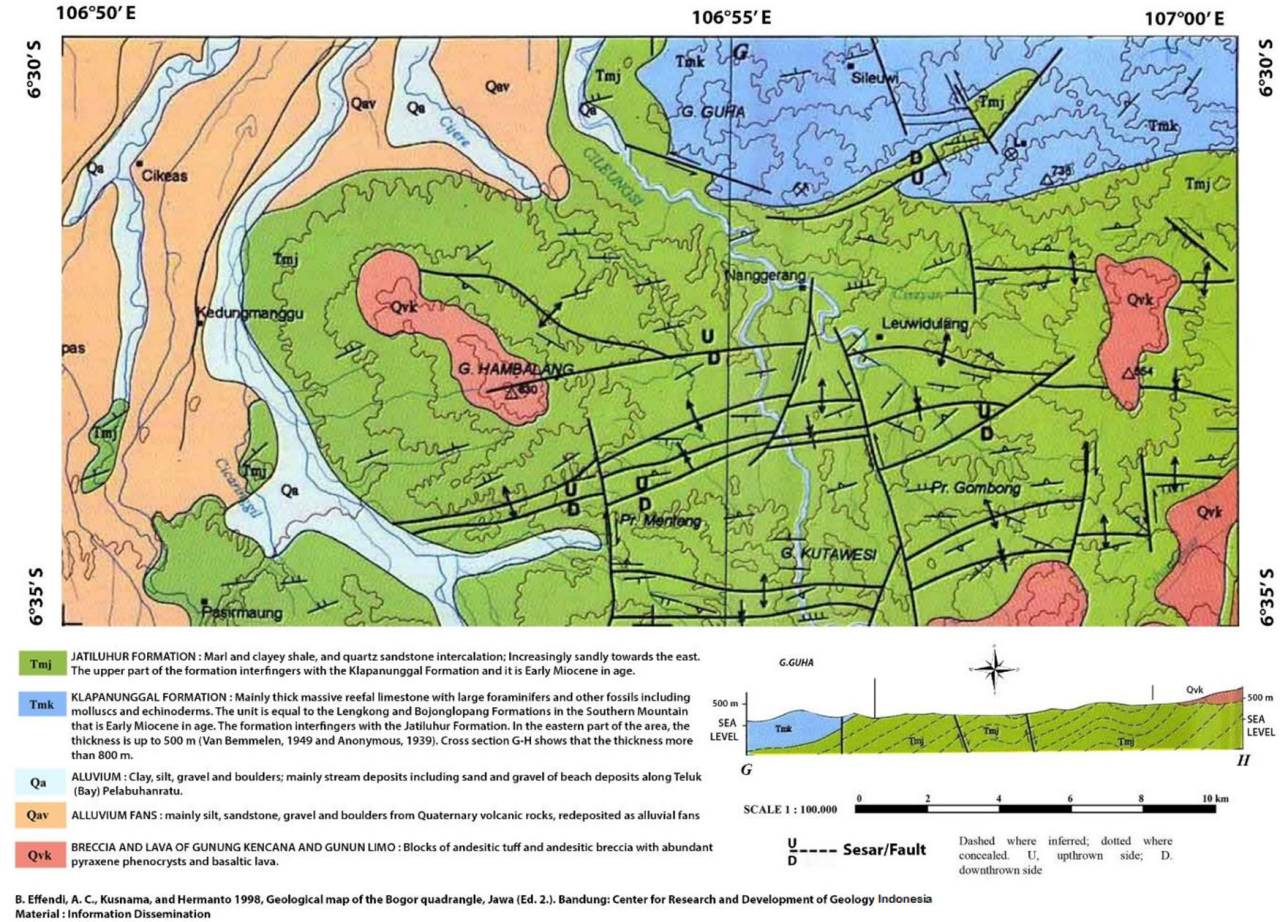
Systematic geological map of Hambalang, Bogor, Indonesia. ^26^ Reproduced with permission from the Head of the Geological Survey Institute of Indonesia.

The type of soil used in this research is soft soil located in *Hambalang, Bogor.* The rock that makes up the area at the top is in the form of Quaternary volcanic breccia; it is less compacted, with its surface utterly weathered into sandy clay. Its colour is gray-brown, it is soft and 0.30-1.50 meters thick. In the breccia unit, the localities have lenses or inserts that are flaky, swollen, stiff, partially scraped, and soft when exposed on the surface. Their colour is gray to brownish-gray. Figure 1 shows a systematic geological map of Bogor, Indonesia. ^26^ The green area is the sampling location used in this study where the surficial deposits show clay shale.

### Experimental technique

The method used in this research is a descriptive method, with tests conducted in the laboratory. Soil samples were taken in October during rainy season near the project site of the homestead of Athlete Hambalang, Bogor, Indonesia. Its geographical coordinates are 6°33′14.5″S and 106°53′22.1″E. Soil sampling was taken from a depth of 1 m to approximately 200 kilograms. Soil materials used for the test were disturbed and undisturbed soil samples. The undisturbed soil sample was used for dry density and engineering properties testing. The test was applied to identify the existing soil. Also, several stages of the laboratory tests were carried out with the disturbed samples. The laboratory test included (i) index properties of soil test, (ii) standard proctor compaction test, and (iii) California Bearing Ratio test (CBR) soaked and unsoaked test. Index properties of soil test included specific gravity, sieve analysis, hydrometer test, and atterberg limit used to determine the classification of soil. Before the compaction and CBR test was started, the soil sample was dried and filtered with sieve number 4 (smaller than 4.75 mm). Also, lime and brick powder were filtered with sieve number 40 (smaller than 0.475 mm). Lime and brick powder were obtained from Bogor, West Java. The additives were mixed as a percentage of 0%, 5%, 10%, and 15% of soft soil ratio by weight. In the Standard Proctor test, each mixture of the additives required 6 samples with the composition of each sample being 2 kg of soil mixed with either lime or brick powder, based on the percentage determined by each sample. Each percentage of the mixture has 6 samples where the total sample is 48 for lime and brick powder. Furthermore, the Standard Proctor test was carried out to obtain optimum moisture content (Wopt) and maximum dry density (γd max) for each mixed sample of the various proportions of lime and brick powder. The CBR test compares the resistance to penetration of the test specimen to that of a standard sample of well-graded crushed stone material using a standard-sized piston in a simple empirical approach. CBR test is one of the ways used to measure the bearing capacity of subgrades. ^27^ Based on the optimum moisture content (Wopt) obtained from the standard Proctor test, we could use the optimum moisture content for the next stage, that is, to conduct the soaked and unsoaked CBR test with various mixtures of the additives. Each mixture of the additives required 2 samples with the composition of each sample being 5 kg of soil mixed with lime or brick powder, based on the percentage determined by each sample. So for each soaked and unsoaked CBR test, 8 samples are needed. CBR tests were carried to study the behavior and bearing capacity of the soil when mixed with the additives (lime and brick powder). After molding the soil shape, each of the soil sample used for the soaked CBR test was soaked in water for 4 days (96 hours), before the penetration test. While in the unsoaked CBR test, no soaking was carried out, but a direct penetration test. The sample was left in the mold to be used for penetration test. The piston was placed on the sample with the perforated plate and the necessary surcharge weights where put in place on the soil. Loading began at a rate of 0.05 in (12.7 mm) per minute. Test loads were recorded at eleven predefined depths of up to 0.500in as the piston entered the soil (13 mm). All these tests are also referred to as ASTM standards. ^28^^–^^33^ Manufacturers of the dial gauge equipment used Mitutoyo analog type.

### Statistical analysis

Statistical analysis using 2nd order polynomial regression was done. The software used was Microsoft Excel 2019. Polynomial relation regression results show quadratic regression function and coefficient of determination (R ^2^). The coefficient of determination is a statistical measurement that shows how much ability the independent variable has in explaining the dependent variable. A value close to 1.0 indicates a regression function that explains a lot of the function variable. This makes it a very reliable model for forecasting the future. While a value close to 0.0 indicates that the calculation fails to simulate the data accurately.

## Results and discussion

The physical and mechanical properties of the original soil can be seen in Table 1. ^36^ The soil samples contained more than 95% fine-grains, with size smaller than 0.075 mm. Based on the results, *Hambalang* soils can be classified as soft soils with high plasticity (symbol MH based on the Unified Soil Classification System) due to their undrained shear strength of >25 kPa. Also, in a previous report, the Department of Settlements and Regional Infrastructure classifies *Hambalang* soil as fat clay because it has a high swelling potential.

**Table 1.** Properties of original soil.

Parameters	Unit	Value
**A. Index properties**		
Moisture Content	%	33.58
Specific Gravity (Gs)	%	2.67
Liquid Limit	%	58.31
Plastic Limit	%	41.78
Plasticity Index	%	16.53
Dry density	kN/m ^3^	1.58
**B. Grain size distribution**		
% Gravel	%	0
% Sand	%	4.55%
% Silt and Clay	%	95.45%
**C. Engineering properties**		
Triaxial UU		
Ҩ	-	12.47
Undrained shear strength	kPa	17.9
**E. Classification**		
USCS	-	MH
**F. Bearing capacity**		
CBR Soaked at (MDD)	%	3.51
CBR Unsoaked at (MDD)	%	15.70

Triaxial UU = unconsolidated undrained test is compression test, in which the soil specimen is subjected under isotropic all-round pressure in the triaxial cell before failure is brought about by increasing the major principal stress. MDD = maximum dry density.

**Figure 2.**
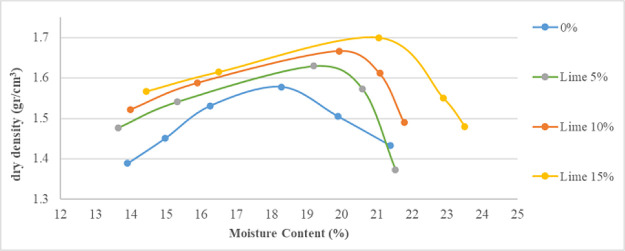
Moisture content vs dry density stabilized with lime.

**Figure 3.**
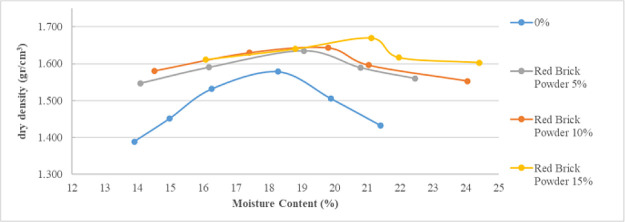
Moisture content vs dry density stabilized with brick powder.

Figures 2 and 3 show the relations between moisture content against the dry density for the various proportions of lime and brick powder obtained from the compaction test. The figures also show the influence of lime and brick powder on the optimum moisture content of the soil.

Lime and brick powder have high effect on the soil's maximum dry density (MDD) in all the mixed samples. The highest is observed in the samples with 15% additive, where the value increased from 1.58 for the soil not mixed to 1.7 for the soil mixed with 15% lime and 1.68 for the soil mixed with 15% brick powder. These were obtained at optimum moisture content of 21.115% for brick powder and 21.06% for lime. Optimum moisture in the compaction test was used as a mixture in the CBR test. The MDD of the soil samples increased because lime and brick powder have relatively higher specific gravities than soil. When the proportion of lime and brick powder increases, the maximum dry density of the soil increases compared to the original soil. The increase in water content at MDD conditions can be seen in Figures 2 and 3. This can occur because of the pozzolanic reaction which causes the maximum density conditions to require a little more water.

**Figure 4.**
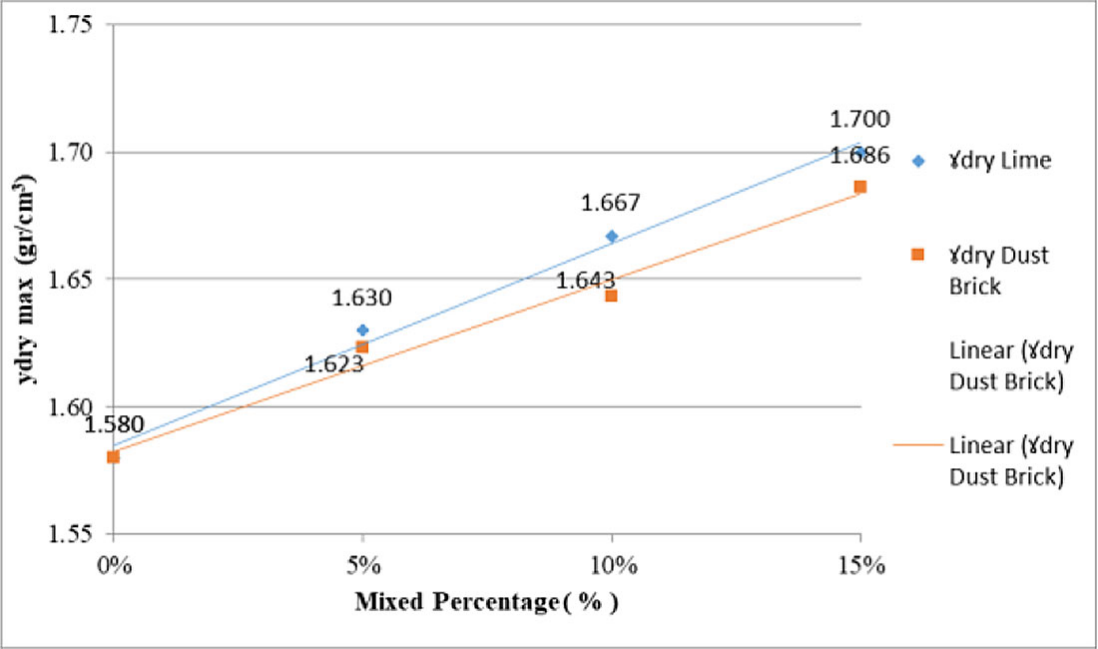
The dry density of the original soil with different additive levels.

As seen in Figure 4, the maximum dry density of the mixture of lime and brick powder is at the 15% additive level. The increase in dry bulk density (γd) was due to additional material filling the cavities in the original soil decreasing the pore number. A decrease in the incidence of an increase in soil density leads to an increase in dry density.

**Figure 5.**
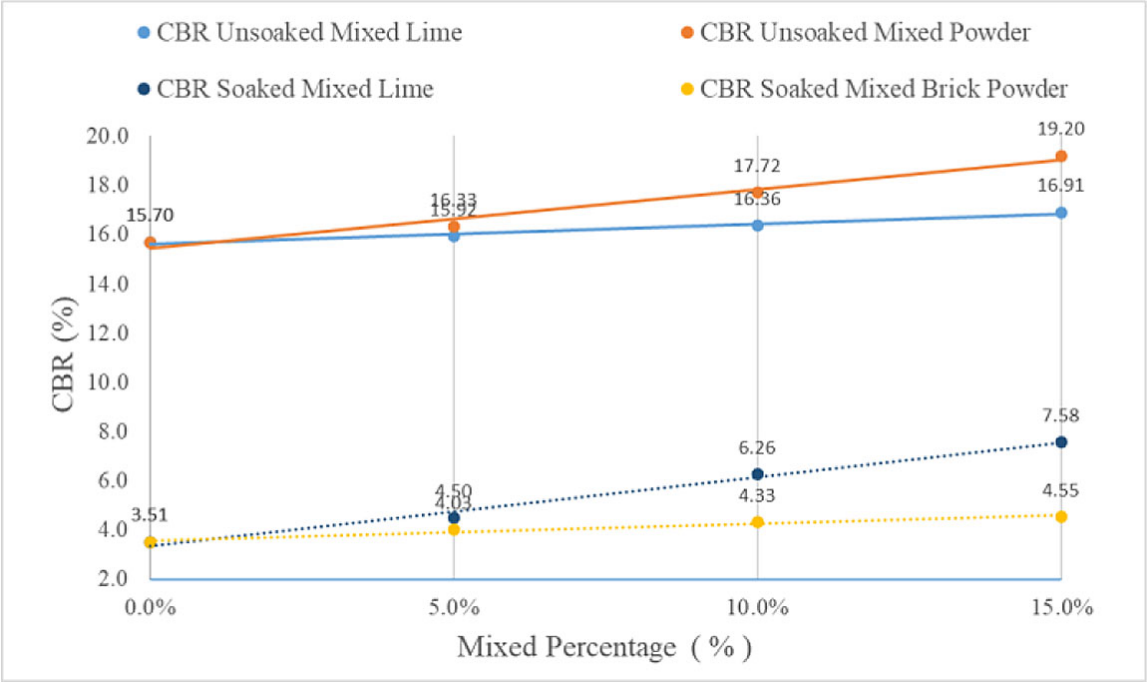
Percentage values of CBR soaked and unsoaked mixed sample.

The CBR soaked and CBR unsoaked tests were carried out with the original soil from lime and red brick powder at predetermined levels (5%, 10%, and 15%) by observing any changes in the CBR value at the top and bottom. The relationship between the addition of lime and red brick powder to the original soil on the CBR values of soaked and unsoaked is shown in Figure 5.

The results of the test data on the discovery of CBR values that were soaked and soil that were not soaked increased with the addition of lime or red brick powder. This is because the original soil grains and additives react with one another in a process known as pozzolanization reaction. In mixed soil samples, the strength generated is determined by the strength of the brick grain and the friction between the grains, because the soil and brick grains are non-cohesive. So, the strength of the mixed soil is not due to cohesion but due to friction between grains. This finding agrees with another finding, which explained the strength of mixed soils in different cases. ^34^

The highest CBR value of lime and red brick powder is found in the 15% mixture. The CBR-soaked result for lime has a higher value (7.58%) than that of brick powder (4.55%). While the CBR unsoaked result of red brick powder has the highest CBR value (19.2%) compared to lime (16.91%).

The CBR soaked value is smaller than the CBR unsoaked value because, at the time of immersion, the water initially fills the pore cavities. Over time, the size of the soil grains expands to their maximum when the water is saturated. In these conditions, the bonds between the soil grains become weak so that the bearing capacity of the soil decreases. This result is similiar to another finding which showed that the more saturated the soil is, the less its bearing capacity. ^35^

The increase in CBR value is due to the cementation process, which makes the soil clump, thus increasing the binding power between the grains. This makes the pore cavity to be surrounded by a more rigid cementation material, which results in the grains becoming strong and not easily destroyed. ^21^

## Conclusions

The MDD of the soil sample increases when mixed in various proportions of lime and brick powder. The soil sample mixed with 15% of additives had the highest maximum dry density. The soaked and unsoaked CBR values increased with the variation of lime and red brick powder. The highest CBR soaked value is obtained from a lime mixture of 15%. The highest unsoaked CBR value can be obtained from a combination of red brick powder by 15%. However, this study has a very large range of variations because of the many material requirements for each test. We suggest other researchers perform the CBR test by reducing the range of variations in the additives to get firm data and using our experimental procedure in this study for further research.

## Data availability

### Underlying data

Zenodo: A comparative study of red brick powder and lime as soft soil stabilizer (Dataset). https://doi.org/10.5281/zenodo.5028251. ^36^

This project contains the following underlying data:

- CBR soaked and unsoaked 10(percent) Mixed with Lime.xlsx
- CBR soaked and unsoaked 15(percent) Mixed with Lime.xlsx
- CBR soaked and unsoaked 5(percent) Mixed with Lime.xlsx
- CBR soaked and unsoaked resume value mixed percentage.xlsx
- CBR soaked and unsoaked soil.xlsx

Data are available under the terms of the Creative Commons Attribution 4.0 International license (CC-BY 4.0).

## Competing interests

No competing interests were disclosed.

## Grant information

This research was supported by UP2M Politeknik Negeri Jakarta through the PNJ DIPA funding [184/PL3.18/SPK/2021]. The funders had no role in study design, data collection, and analysis, decision to publish, or preparation of the manuscript.
