# Supplementary material for: A comparative study of red brick powder and lime as soft soil stabilizer
Source: F1000Res. 2022 Jul 20;10:777. Originally published 2021 Aug 9. [Version 2] doi: 10.12688/f1000research.27835.2 (PMC10172792; doi:10.12688/f1000research.27835.2)
Supplement: Peer review history

# Peer review: Reviewer response for version 2

**DOI:** 10.5256/f1000research.136027.r171917

**Published:** 2023-05-22

**Authors:** Chinchu Cherian

**Affiliations:** 1 The University of British Columbia, Vancouver, British Columbia, Canada

1. General comments:

- The article titled "A comparative study of red brick powder and lime as soft soil stabilizer", authored by Salimah *et al.*, presents a laboratory study conducted to understand the effects of lime or red brick powder additives on a soft soil stabilization. In general, the topic is well within the scope of the journal, and the authors have provided a clear rationale or justification for conducting this study. However, there have been relevant prior studies performed on this topic of interest.

2. Literature review:

- Although the study is relevant, there is ample amount of research work done in this topic before. The authors have missed many such studies while discussing the literature review. It is recommended to incorporate these articles and provide a more critical summary of the literature findings and thereby the important knowledge gap addressed in this study. 

3. Objectives and scope of work:

- This study has made significant contributions by considering both undisturbed and disturbed soil samples. They also used statistical analysis for the data interpretation. The authors have clearly defined the objectives and outlined the experimental methods.

4. Results and discussion: 

- The authors have performed an in-depth analysis of the obtained experimental data and discussed the major results/findings with ample reasons, particularly in a manner that is both comprehensive and accessible to readers. 
- It is however recommended to perform some additional analytical tests on the treated soil samples (like SEM and XRD) to comprehend the chemical changes that led to the mechanical improvement of the soil.

5. Conclusions: 

- Although the authors wrote a clear summary and conclusions of the study, the reviewer feels that the implications of this study for future research and practical applications is missing. 

Is the work clearly and accurately presented and does it cite the current literature?

Partly

If applicable, is the statistical analysis and its interpretation appropriate?

I cannot comment. A qualified statistician is required.

Are all the source data underlying the results available to ensure full reproducibility?

Yes

Is the study design appropriate and is the work technically sound?

Partly

Are the conclusions drawn adequately supported by the results?

Yes

Are sufficient details of methods and analysis provided to allow replication by others?

Yes

Reviewer Expertise:

Geotechnics, Soil stabilization, Material characterization, Solid waste treatment and upcycling, Roads and Infrastructure development, Green construction

I confirm that I have read this submission and believe that I have an appropriate level of expertise to confirm that it is of an acceptable scientific standard, however I have significant reservations, as outlined above.

# Peer review: Reviewer response for version 2

**DOI:** 10.5256/f1000research.136027.r171916

**Published:** 2023-05-10

**Authors:** Hamid Gadouri, Brahim Meziani

**Affiliations:** 1 Geomaterials Laboratory, Civil Engineering Department, Universite Hassiba Benbouali de Chlef, Chlef, Chlef Province, Algeria; 2 Earth Sciences Department, Universite de Khemis Miliana, Khemis Miliana, Aïn Defla Province, Algeria

**1. General comments:**

 The article titled "A comparative study of red brick powder and lime as soft soil stabilizer", authored by Aisyah Salimah *et al*., presents a practical study aimed at understanding the laboratory tests with disturbed and undisturbed soil samples where the proportions of lime or red brick powder additives are 0% up to 15% of the soil sample. In general, the topic is of great interest and is well within the scope of the journal. The authors have provided a significant contribution that is sufficiently performed and interesting for readers. They used an appropriate method to address the objectives, and the obtained results are both interesting and relevant. The findings were successfully explained in the manuscript, and most of the references used are related to the objective of the work. The scientific contribution of this paper highly meets the requirements of the journal F1000Research.

**2. Abstract section:**

 The abstract effectively provides a brief overview of the study's background, methods, and results. The language used is clear and concise, with technical terms explained where necessary, making it easy to understand for readers without prior knowledge of the topic. The abstract follows a structured approach, starting with the study's background and objectives, followed by the methods and results, and ending with conclusions. This helps readers quickly understand the main purpose and outcomes of the study. Overall, the abstract is well-written and effectively conveys the main findings of the study.

**3. Introduction section:**

 In the introduction section of this scientific paper, the author extensively discussed the characteristics of soft soil with weak geotechnical properties, as well as the importance of studying it before undertaking any civil engineering project. The author touched on the most important numerical models developed by researchers regarding geotechnical projects in general, and shallow tunnels modeling in particular, based on studies conducted and published in relevant scientific journals. The author relied on several important references related to the subject matter. The introduction is clear in terms of linguistic structure, where we find that the problem is completely clear.

**4. Methods section:**

 The authors conducted a rigorous experiment using a variety of tests to assess the effectiveness of the two different stabilizers. Their findings suggest that the method used in the study is not only appropriate but also highly effective in improving the strength and stability of soft soil, which has important implications for construction projects in areas with problematic soil conditions." This sentence highlights the fact that the authors used a rigorous approach in their study, which involved various tests to assess the effectiveness of the stabilizers. Additionally, the sentence emphasizes that the method used is not only appropriate but also highly effective, which is a strong endorsement of the validity of the research findings. Finally, the sentence suggests that the implications of the research are significant for construction projects in areas with problematic soil conditions, which adds further weight to the importance of the study. 

 The statistical analysis performed on the moisture content and dry density data was useful, which indicates that the use of 2nd order polynomial regression with Microsoft Excel 2019 software is a meticulous approach to the data analysis process. The inclusion of the coefficient of determination (R2) is noteworthy as it provides crucial insight into the explanatory power of the independent variable with respect to the dependent variable. The successful calculation of the quadratic regression function and R2 value highlights a significant correlation between the two variables, making the regression model a dependable tool for predicting future trends. In conclusion, this “statistical analysis” establishes a robust foundation for the statistical analysis of the data, and the results can be interpreted confidently to derive meaningful insights.

**5. Results and discussion section:**

 That said, the article is a well-written and informative piece of research that successfully presents the findings of the study. The authors have clearly outlined their methodology and have presented the results in a manner that is both comprehensive and accessible to readers. Overall, the article is a valuable contribution to the field of soft soil stabilization and provides important insights into the effectiveness of different stabilizers in enhancing soil strength and stability." The authors provide a detailed discussion section that highlights the significance of their findings and their implications for future research and practical applications. The discussion section begins by summarizing the key results of the study, emphasizing the effectiveness of both red brick powder and lime as soft soil stabilizers. The authors then provide an in-depth analysis of their findings, discussing the various factors that may have contributed to the effectiveness of the stabilizers, including their chemical composition and particle size. In addition to discussing their own research, the authors also reference previous studies on soft soil stabilization, providing a broader context for their findings and highlighting areas where further research is needed. They also discuss the practical applications of their research, such as the potential use of red brick powder and lime in construction projects to improve the stability and safety of buildings and infrastructure in areas with problematic soil conditions. Overall, the discussion section of the article is well-written and thorough, providing a detailed analysis of the study's findings and their implications for the field of soft soil stabilization. The authors' ability to contextualize their research within existing literature and to provide practical applications for their findings is a testament to the strength of the study and the potential impact it could have on future research and real-world applications.

**6. Conclusion section:**

 Based on this section, the authors have effectively summarized the key findings of the study in a concise and clear manner. The conclusion begins by stating the aim of the study, which is to investigate the effectiveness of lime and red brick powder as soft soil stabilizers. The authors then highlight the various laboratory tests that were conducted to evaluate the performance of the stabilizers, including Specific gravity, Sieve analysis, Hydrometer test, Atterberg limit, Proctor test, and CBR. The authors then proceed to summarize the key findings of the study, noting that the addition of lime and red brick powder to soft soil samples resulted in an increase in MDD (Maximum Dry Density) and CBR (California Bearing Ratio). They further note that the highest values of MDD and CBR were obtained with a 15% addition of the respective stabilizer. The authors provide specific percentages of increase in CBR values over the untreated soil for both soaked and unsoaked conditions, which suggests a significant improvement in soil strength with the addition of stabilizers. Overall, the conclusion section effectively highlights the key results of the study and their significance for the field of geotechnical engineering. The authors have effectively communicated their findings in a clear and concise manner, which adds to the strength and clarity of the article.

 However, based on the above comments, the present paper “A comparative study of red brick powder and lime as soft soil stabilizer” can be Approved for indexing as it is

Is the work clearly and accurately presented and does it cite the current literature?

Yes

If applicable, is the statistical analysis and its interpretation appropriate?

Yes

Are all the source data underlying the results available to ensure full reproducibility?

Yes

Is the study design appropriate and is the work technically sound?

Yes

Are the conclusions drawn adequately supported by the results?

Yes

Are sufficient details of methods and analysis provided to allow replication by others?

Yes

Reviewer Expertise:

Geotechnical Engineering and Soil Improvement

We confirm that we have read this submission and believe that we have an appropriate level of expertise to confirm that it is of an acceptable scientific standard.

# Peer review: Reviewer response for version 2

**DOI:** 10.5256/f1000research.136027.r144746

**Published:** 2022-08-08

**Authors:** Reginald B. Kogbara

**Affiliations:** 1 Department of Environmental Engineering, İzmir Institute of Technology, İzmir, Turkey; 2 Mechanical Engineering Program, Texas A&M University at Qatar, Doha, Qatar

The article has been satisfactorily revised.

Is the work clearly and accurately presented and does it cite the current literature?

Partly

If applicable, is the statistical analysis and its interpretation appropriate?

Yes

Are all the source data underlying the results available to ensure full reproducibility?

Yes

Is the study design appropriate and is the work technically sound?

Yes

Are the conclusions drawn adequately supported by the results?

Yes

Are sufficient details of methods and analysis provided to allow replication by others?

Yes

Reviewer Expertise:

Stabilization/solidification of problematic soils and wastes, subgrade soil stabilization using polymers, characterization, and utilization of cement-based materials.

I confirm that I have read this submission and believe that I have an appropriate level of expertise to confirm that it is of an acceptable scientific standard.

# Peer review: Reviewer response for version 1

**DOI:** 10.5256/f1000research.30780.r138934

**Published:** 2022-06-22

**Authors:** Reginald B. Kogbara

**Affiliations:** 1 Department of Environmental Engineering, İzmir Institute of Technology, İzmir, Turkey; 2 Mechanical Engineering Program, Texas A&M University at Qatar, Doha, Qatar

The paper concisely describes experiments aimed at investigating the effect of lime and red brick powder on soft soil stabilization. Specifically, its use of red brick powder for soil stabilization allows for beneficial reuse of what might otherwise be a waste material for soil stabilization. Hence, the work is scientifically valid and contributes to knowledge in the area of soil stabilization. The experimental design is appropriate and the work is technically sound with sufficient details that allow replication of the work by others, and the conclusions made are supported by the results of the study.

**Major Points:**

- The authors should better put the work into context by citing current literature that deal with similar investigations and highlighting the contribution of this work that is different from what was done in similar studies. For example, see Blayi *et al.,* (2020) ^1^; Srikanth Reddy *et al*., (2018) ^2^; and Bhavsar *et al*., (2014) ^3^
- Could the authors please state the sources of the lime and red brick powder used for the soil stabilization?

**Minor Points:**

- It would have been more helpful if compressive strength data was included to facilitate comparison with data in other studies related to the use of lime and waste brick powder for soil stabilization.
- The authors should explain clearly the expression “Statistical analysis using 2nd order polynomial regression was done”, stating the specific dataset the polynomial regression was applied on. For example, was the polynomial regression done on the moisture content – dry density data?

Is the work clearly and accurately presented and does it cite the current literature?

Partly

If applicable, is the statistical analysis and its interpretation appropriate?

Yes

Are all the source data underlying the results available to ensure full reproducibility?

Yes

Is the study design appropriate and is the work technically sound?

Yes

Are the conclusions drawn adequately supported by the results?

Yes

Are sufficient details of methods and analysis provided to allow replication by others?

Yes

Reviewer Expertise:

Stabilization/solidification of problematic soils and wastes, subgrade soil stabilization using polymers, characterization, and utilization of cement-based materials.

I confirm that I have read this submission and believe that I have an appropriate level of expertise to confirm that it is of an acceptable scientific standard, however I have significant reservations, as outlined above.

## References

[1] : Stabilization of high-plasticity silt using waste brick powder. *SN Applied Sciences* .2020;2(12) : 10.1007/s42452-020-03814-8 10.1007/s42452-020-03814-8

[2] : Lime-Stabilized Black Cotton Soil and Brick Powder Mixture as Subbase Material. *Advances in Civil Engineering* .2018;2018: 10.1155/2018/5834685 1-5 10.1155/2018/5834685

[3] : EFFECT OF BURNT BRICK DUST ON ENGINEERING PROPERTIES ON EXPANSIVE SOIL. *IJRET* .2014;3(4) : Reference source

# Peer review: Reviewer response for version 1

**DOI:** 10.5256/f1000research.30780.r91503

**Published:** 2021-11-10

**Authors:** A. U. Ravi Shankar

**Affiliations:** 1 Department of Civil Engineering, National Institute of Technology Karnataka, Mangalore, Karnataka, India

The authors have conducted laboratory investigation to improve CBR of silty soil by using red brick powder and lime. The authors stabilized fine grain soil with and brick powder. The CBR values improved up 15 % addition of red brick powder. They could satisfy their objective. Not much information is generated. The reason for improvement in strength. The chemical composition of Brick Powder, Gradation etc. are missing. If it is only for pavement then only CBR improvement may not be sufficient as per new codes. Within the presented data the following pros and cons are observed. The suggested modification along with grammar check may help in improving the quality of paper.

 The Text should be written in third person - 'I' and 'we' should not be there.

**Abstract:**

 Statement: Soil has an important role to play in planning buildings because it supports the loads above it.

- Comment: May be corrected as 'Role of soil in building construction...'.

Statement: Soil stabilization can be defined as the process of stabilizing soil properties by chemical or physical means to improve its engineering efficiency.

- Comment: Can be corrected as ‘process of modifying the soil properties....’.

**Introduction:**

 Statement: Therefore, it is necessary to do more research on the effect of red brick powder on soil stability. Also, lime and red brick powder are compared in this work. The impact of lime and red brick powder on the geotechnical qualities of soft soil was examined by conducting the CBR test.

- Comment: Modify the sentences as ‘Also, the stabilizing effect of lime and red brick powder are compared in this work. The influence of lime and red brick powder on the geotechnical qualities of soft soil was examined by conducting the CBR test'.

Statement: The CBR value increased because the soil structure changed from being dispersed to flocculated. Thus, in this study, lime and brick powder were used as stabilizers on soft soils.

- Comment: Remove the above statements.

Statement: In this study, the index and engineering properties of the original soil and mixed soil were tested.

- Comment: Remove the term ‘original’ and may be replaced with term either ‘soil or silty soil or untreated soil’ wherever applicable in the manuscript.

Statement: Literature review

- Comment: Literature review can be combined with introduction part.

**Methods:**

 Statement: The proportions of lime or red brick powder additives mixed together are 0%, 5%, 10%, and 15% of the original soil sample.

- Comment: Can be corrected as 'The proportions of lime or red brick powder additives mixed are 0%, 5%, 10%, and 15% with the soil'.

Statement: From the results of the laboratory tests, the soil type obtained is MH soil based on the Unified Soil Classification System (USCS). The MH soil type is a low plasticity silt soil.

- Comment: Can be corrected as ‘The soil type obtained is MH (low plasticity silt) as per the Unified Soil Classification System (USCS)'.

**Material and sampling:**

- Comment: Properly described.

**Experimental technique:**

 Statement: proctor, atterberg

- Comment: Capitalize first letter as ‘Proctor’, ‘Atterberg’

Statement: CBR, and other abbreviations

- Comment: All abbreviations are to be used at the first appearance throughout the manuscript.

**Results and discussion:**

 Statement: However, red brick powder had a significant increase of 15%.

- Comment: Can be corrected as ‘However, red brick powder addition has contributed to a significant increase in CBR value (15%)'.

Statement: Table 1

- Comment: Atterberg limits, CBR need to be indicated without decimals. Moisture contentment can be represented with 1 decimal. For Parameter % sand, unit %, value 4.55%, include correction as - Parameter sand, unit %, value 4.55. Make corrections wherever applicable in the manuscript.

Statement: Figure 2, 3 and 4

- Comment: Check x and y axes titles and units and incorporate correction (uniform writing style and standard units are to be used).

Statement: Lime and brick powder have high effect on the soil's maximum dry density (MDD) in all the mixed samples.

- Comment: Instead of term ‘high effect’ use ‘significant' or 'notable effect’.

Statement: Figure 5

- Comment: Correction to be incorporated for CBR values.

Statement: Description of Results and discussion.

- Comment: Description has grammatical errors. It could be improved. Needs modification.

**Conclusions:**

 Statement: This study found a very large range of variations because of the many material requirements for each test. We suggest other researchers perform the CBR test by reducing the range of variations in the additives to get firm data and using our experimental procedure in this study for further research.

- Comment: Authors need to draw conclusions on your work. Suggestions are not needed. Rewrite conclusions.

Is the work clearly and accurately presented and does it cite the current literature?

Yes

If applicable, is the statistical analysis and its interpretation appropriate?

Yes

Are all the source data underlying the results available to ensure full reproducibility?

Yes

Is the study design appropriate and is the work technically sound?

Yes

Are the conclusions drawn adequately supported by the results?

Yes

Are sufficient details of methods and analysis provided to allow replication by others?

Yes

Reviewer Expertise:

Pavement Materials, Concrete Pavement

I confirm that I have read this submission and believe that I have an appropriate level of expertise to confirm that it is of an acceptable scientific standard, however I have significant reservations, as outlined above.
